# Prevalence and Incidence of Fractures in Patients With Nonfunctional Adrenal Tumors

**DOI:** 10.1001/jamanetworkopen.2024.6453

**Published:** 2024-04-15

**Authors:** Jonatan D. Lindh, Jekaterina Patrova, Buster Mannheimer, Henrik Falhammar

**Affiliations:** 1 Department of Laboratory Medicine, Division of Clinical Pharmacology, Karolinska Institutet, Stockholm, Sweden; 2 Department of Clinical Science and Education, Södersjukhuset, Karolinska Institutet, Stockholm, Sweden; 3 Department of Endocrinology, Södersjukhuset, Stockholm, Sweden; 4 Department of Molecular Medicine and Surgery, Karolinska Institutet, Stockholm, Sweden; 5 Department of Endocrinology, Karolinska University Hospital, Stockholm, Sweden

## Abstract

**Importance:**

It is unclear whether nonfunctional adrenal tumors (NFATs) are associated with fractures.

**Objective:**

To analyze fracture frequencies in individuals with NFATs.

**Design, Setting, and Participants:**

A national retrospective cohort study was conducted in patients with NFATs diagnosed in Sweden between January 1, 2005, and December 31, 2019, and control participants without adrenal tumors followed up until death or the end of 2019. Individuals with a diagnosis of adrenal hormonal excess or previous malignant tumors were excluded. Sensitivity analyses were performed in subgroups of individuals with a combination of gallbladder, biliary tract, and pancreas diseases (for whom it was assumed that controls would also have undergone computed tomography) and 3- and 12-month survival free of malignant tumors after the NFAT diagnosis. The data were analyzed from September to November 2023.

**Exposures:**

Diagnosis of NFATs.

**Main Outcomes and Measures:**

Main study outcomes were prevalence and incidence of fractures after adjustment for sex, age, and comorbidities. Secondary outcomes were fragility fractures, fractures with fall on the same level, and fracture locations (distal arm and vertebral and hip fractures). Fracture incidence after adrenalectomy was also studied.

**Results:**

Among 20 390 patients, 12 120 (59.4%) were women, and the median (IQR) age was 66 (57-73) years; among 125 392 controls, 69 994 (55.8%) were women, and the median (IQR) age was 66 (57-73) years. Previous fractures were more common in patients diagnosed with NFATs compared with controls (4310 of 20 390 [21.1%] vs 20 323 of 125 392 [16.2%]; odds ratio [OR], 1.39; 95% CI, 1.34-1.45; adjusted OR [AOR], 1.27; 95% CI, 1.23-1.33). During the follow-up period (median [IQR], 4.9 [2.2-8.2] years), incident fractures were more common in patients with NFATs (3127 of 20 390 [15.3%] vs 16 086 of 125 392 [12.8%]; hazard ratio [HR], 1.40; 95% CI, 1.34-1.45; adjusted HR [AHR], 1.27; 95% CI, 1.22-1.33). An association between NFATs and vertebral fractures was found (AOR, 1.51; 95% CI, 1.33-1.72; AHR, 1.83; 95% CI, 1.60-2.09). In men younger than 50 years, NFATs were associated with fractures (AOR, 1.45; 95% CI, 1.21-1.74; AHR, 1.48; 95% CI, 1.20-1.82). There was no association among individuals who had undergone adrenalectomy (AHR, 1.12; 95% CI, 0.90-1.38). The association between NFATs and fractures remained significant and of similar magnitude in all sensitivity analyses.

**Conclusions and Relevance:**

In this cohort study, NFATs were associated with fractures, particularly among younger men; thus, patients with NFATs should have bone health evaluation with appropriate treatment and monitoring, especially in younger men.

## Key Points

**Question:**

Are fracture incidence and prevalence among patients with nonfunctional adrenal tumors (NFATs) higher than in the general population?

**Findings:**

In this cohort study of 20 390 patients with NFATs and 125 392 controls, both the prevalence of previous fractures and incidence of new fractures during a median follow-up period of 4.9 years were higher among patients with NFATs compared with controls.

**Meaning:**

Fractures were more common in patients with NFATs than in controls; thus, bone health evaluation with appropriate treatment and monitoring should be performed, particularly in younger men with NFATs.

## Teaser

This cohort study analyzes fracture prevalence and incidence in patients with nonfunctional adrenal tumors in Sweden compared with controls.

## Introduction

The advancement and increased use of abdominal imaging, such as computed tomography and magnetic resonance imaging, has resulted in an increased detection of adrenal tumors.^1,2,3^ The mean prevalence of adrenal tumors in the general population is approximately 1.4% but 1.9% to 3.2% in those older than 65 years.^4,5^ An adrenal incidentaloma is an adrenal tumor that has been detected on imaging performed for reasons other than suspected adrenal disease or cancer staging.^3^

Benign nonfunctional adrenal tumors (NFAT) are the great majority of all adrenal incidentalomas and display no overt hormonal secretion.^3^ However, mild autonomous cortisol secretion (MACS; ie, overproduction of small quantities of cortisol without overt Cushing syndrome, previously known as subclinical Cushing syndrome) is common.^3^ Using the most recent European guidelines, which define MACS as a serum cortisol concentration after administration of 1 mg of dexamethasone of greater than 1.8 μg/dL (>50 nmol/L) without clinical signs of overt Cushing syndrome,^3^ studies have shown a prevalence of 43% to 45% in adrenal incidentalomas.^6,7,8^ A previous long-term study found a conversion from NFAT to MACS of 0% to 31%.^9^ MACS has been reported to be associated with mainly increased cardiometabolic risk,^10^ but increased occurrences of fractures have also been found in studies.^11,12^ Interestingly, in a population-based cohort study conducted in 1 county, patients with NFAT were more likely to have previous fractures when diagnosed with an NFAT and an increased incidence of new fractures during follow-up.^13^ However, larger studies are needed. The aim of this study was to analyze fracture prevalence and incidence during follow-up in all patients with NFATs in Sweden compared with controls. Subgroup analyses were also planned to better characterize the risk of fractures.

## Methods

### Study Design and Setting

This cohort study was approved by the Swedish Ethical Review Authority, which did not require informed consent due to the retrospective nature of the study. Part of this cohort has been used in previous studies.^14,15,16^ The study followed the Strengthening the Reporting of Observational Studies in Epidemiology (STROBE) reporting guideline for cohort studies.

This population-based, retrospective cohort study was conducted in patients with NFATs diagnosed in Sweden between January 1, 2005, and December 31, 2019. The Swedish personal identity number was used for data linkage among several national registers. The National Patient Register, containing all inpatient and specialist outpatient care, was used to identify *International Statistical Classification of Diseases and Related Health Problems, Tenth Revision* (*ICD-10*) codes from 1997 until 2019. The Cause of Death Register was used to identify individuals who had died between 2005 and 2020. Drug dispensations were identified using the Anatomical Therapeutic Chemical (ATC) codes and the Swedish Prescribed Drug Register from mid-2005 until 2019. All individuals with a first-ever *ICD-10* code of D44.1 (neoplasm of uncertain behavior of adrenal gland) and/or D35.0 (benign neoplasm of adrenal gland) between 2005 and 2019 were identified, and the index date for each individual was set as the date of the first of 1 of these diagnoses. The Total Population Register was used to randomly select age- and sex-matched controls at a 4:1 ratio. Any individuals with a known malignant tumor of any kind (ie, any *ICD-10* C code) diagnosed since 1997 up to the index date were excluded. Any patients with a diagnosis of hormonal activity, such as Cushing syndrome (*ICD-10* code E24), congenital adrenal hyperplasia (*ICD-10* code E25),^14^ primary aldosteronism (*ICD-10* code E26), and pheochromocytoma (*ICD-10* code E27.5), from 1997 until 2019 were excluded from both groups. By excluding all hormonally active lesions in the current study, we focused on only NFATs. However, some cases of MACS may have been included because they may not have received the *ICD-10* code E24. Fractures, osteoporosis medications (antiresorptive and anabolic medications), and confounding factors were identified using the *ICD-10* and ATC codes (eTable 1 in Supplement 1). Fragility fractures were defined as distal arm, vertebral, or hip fractures. Given that many fragility fractures occur during a fall on the same level, these fractures were also identified. The cohort was stratified by sex and at 50 years of age because menopause usually commences at approximately 50 years of age in women.

Because we were not able to assemble a control group limited to persons who underwent abdominal imaging before the index date, we tried to overcome this limitation by conducting a sensitivity analysis. Patients with NFATs and controls with a diagnosis of gallbladder, biliary tract, and pancreas diseases (*ICD-10* codes K80-87) were identified because it can be presumed that both patients with NFATs and controls had undergone imaging. Patients with NFATs were included if they had a K80 to K87 diagnosis within 6 months before the D41.1 and/or D35.0 diagnosis. In the sensitivity analyses, the index date for controls was defined by the date of the first-ever K80 to K87 diagnosis.

In 2 other sensitivity analyses, the index date was moved forward 90 or 365 days after the first D44.1 or D35.0 diagnosis to exclude patients who underwent imaging due to a suspected malignant tumor, which was then confirmed shortly after the NFAT diagnosis. Moreover, we wanted to make the NFATs more representative of nonfunctioning benign adrenal incidentalomas. In controls, the index date was shifted in a similar manner as patients with NFATs in the 2 analyses.

### Statistical Analysis

Descriptive statistics included numbers (percentages) and medians (IQRs), and baseline characteristics were compared between patients with NFATs and controls by means of χ^2^ and unpaired, 2-tailed *t* tests, as appropriate. Fractures during follow-up were presented using Kaplan-Meier curves. Patients with NFATs and controls were compared regarding any fracture (primary outcome) as well as fragility fractures, fractures associated with fall on the same level, fracture locations (distal arm, vertebrae, or hip), and dispensation of osteoporosis medication (secondary outcomes) using logistic regression analysis to evaluate fracture events preceding the index date. Cox proportional hazards regression analysis was used to evaluate events during follow-up. Both analyses were performed with and without adjustment for age, sex, and all other potential confounders specified in eTable 1 in Supplement 1, including previous fractures in the latter analysis . Moreover, analysis of the main outcome was repeated in subgroups based on sex, age, and whether adrenalectomy had been performed. All statistical tests were 2-sided, and *P* < .05 indicated statistical significance. Statistical analysis was conducted in R, version 4.2.2 (R Project for Statistical Computing). The data were analyzed from September to November 2023.

## Results

### Study Population

A total of 20 390 patients with NFATs (12 120 females [59.4%] and 8270 males [40.6%]; median [IQR] age, 66 [57-73] years) and 125 392 controls (69 994 [55.8%] females and 55 398 [44.2%] males; median [IQR] age, 66 [57-73] years) were studied (eFigure in Supplement 1). The most common comorbidities in both patients with NFATs and controls were diabetes and ischemic heart disease. Approximately 1 in 10 had been receiving long-term glucocorticoid treatment, and previous hospitalization was common in both groups. Table 1 provides a selection of medical conditions and socioeconomic factors found in the study population at the index date. eTable 2 in Supplement 1 lists comorbidities affecting bone mineral density (BMD) by sex among patients with NFATs and controls.

**Table 1.** Medical Characteristics and Socioeconomic Factors Among Patients With NFATs and Controls at Baseline^a^

Characteristic	Patients with NFATs (n = 20 390)	Controls (n = 125 392)
Sex		
Female	12 120 (59.4)	69 994 (55.8)
Male	8270 (40.6)	55 398 (44.2)
Age, median (IQR), y	66 (57-73)	66 (57-73)
Diagnosis		
Ischemic heart disease	2779 (13.6)	11 511 (9.2)
COPD	1797 (8.8)	3389 (2.7)
Chronic heart failure	1428 (7.0)	4469 (3.6)
Cerebrovascular diseases	1343 (6.6)	5837 (4.7)
Thrombosis	847 (4.2)	2373 (1.9)
Kidney diseases	587 (2.9)	1823 (1.5)
Liver diseases	379 (1.9)	1000 (0.8)
Inflammatory bowel disease	419 (2.1)	1384 (1.1)
Celiac disease or malabsorption	97 (0.5)	486 (0.4)
Alcohol misuse	1006 (4.9)	3245 (2.6)
Hyperparathyroidism	242 (1.2)	451 (0.4)
Hyperthyroidism	378 (1.9)	1358 (1.1)
Vitamin D deficiency	16 (0.08)	94 (0.07)
Malnutrition	10 (0.05)	26 (0.02)
Hypogonadism^b^	41 (0.2)	149 (0.1)
Diabetes	3017 (14.8)	9660 (7.7)
Previous fractures		
Total	4310 (21.1)	20 323 (16.2)
With fall on the same level	2208 (10.8)	10 524 (8.4)
Fragility fracture		
Total	2165 (10.6)	10 448 (8.3)
With fall on the same level	1233 (6.0)	6053 (4.8)
Distal arm fracture	952 (4.7)	5046 (4.0)
Vertebral fracture	311 (1.5)	1115 (0.9)
Hip fracture	456 (2.2)	2162 (1.7)
Medications		
Glucocorticoids >90 d	2485 (12.2)	10 018 (8.0)
Osteoporosis medications	1165 (5.7)	5404 (4.3)
Previous hospitalization >3 d	11 174 (54.8)	44 344 (35.4)
Socioeconomic factors		
Highest educational level		
Primary and secondary		
<9 y	4205 (20.6)	23 389 (18.7)
9-10 y	2343 (11.5)	11 903 (9.5)
Upper secondary		
<2 y	6847 (33.6)	37 124 (29.6)
3 y	2316 (11.4)	15 665 (12.5)
Postsecondary		
<3 y	1870 (9.2)	14 512 (11.6)
≥3 y	2234 (11)	18 850 (15.0)
Postgraduate	111 (0.5)	1271 (1.0)
Missing	464 (2.3)	2673 (2.1)
Annual income, median (IQR), SEK	162 600 (124 600-239 200)	179 800 (130 100-268 700)
Unemployment, median (IQR), d	0	0

Abbreviations: COPD, chronic obstructive pulmonary disease; NFAT, nonfunctioning adrenal tumor; SEK, Swedish krona (1 SEK = 0.096 US dollars as of March 18, 2024).

^a^ Data are presented as number (percentage) of participants unless otherwise indicated. All variables except celiac disease or malabsorption and vitamin D deficiency differ significantly between cases and controls.

^b^ Not including normal menopause.

### Previous Fractures and Osteoporosis Medications

By using logistic regression analysis, we analyzed previous fractures and osteoporosis medications adjusted for potential confounders (Table 2). Previous fractures were more common in those with NFATs than in controls (4310 of 20 390 [21.1%] vs 20 323 of 125 392 [16.2%]; odds ratio [OR], 1.39; 95% CI, 1.34-1.44; adjusted OR [AOR], 1.27; 95% CI, 1.22-1.32) in the population as a whole as well as among both men and women when analyzed separately. Similarly, NFATs were also associated with previous fractures with a fall on the same level (AOR, 1.18; 95% CI, 1.12-1.24). In both those younger and those older than 50 years, NFATs were associated with previous fractures in both sexes.

**Table 2.** Odds of Fractures and Osteoporosis Medications in Individuals With Nonfunctioning Adrenal Tumors at Baseline Compared With Controls

	Full cohort			Aged <50 y			Aged ≥50 y		
	All (N = 20 390)	Women (n = 12 120)	Men (n = 8270)	All (N = 2349)	Women (n = 1413)	Men (n = 936)	All (n = 18 041)	Women (n = 10 707)	Men (n = 7334)
Age, median (IQR), y	66 (57-73)	66 (57-73)	65 (57-72)	43 (37-47)	43 (37-47)	43 (36-47)	67 (60-74)	68 (61-74)	67 (60-73)
**Previous fractures**									
OR (95% CI)	1.39 (1.34-1.44)	1.31 (1.25-1.37)	1.47 (1.39-1.57)	1.56 (1.38-1.77)	1.56 (1.31-1.86)	1.58 (1.32-1.88)	1.37 (1.32-1.43)	1.29 (1.23-1.36)	1.47 (1.38-1.56)
AOR (95% CI)	1.27 (1.22-1.32)	1.21 (1.15-1.27)	1.34 (1.26-1.43)	1.47 (1.29-1.67)	1.49 (1.24-1.78)	1.45 (1.21-1.74)	1.26 (1.21-1.31)	1.21 (1.15-1.27)	1.33 (1.25-1.42)
With fall^a^									
OR (95% CI)	1.33 (1.26-1.39)	1.26 (1.19-1.33)	1.40 (1.28-1.54)	1.45 (1.15-1.81)	1.32 (0.96-1.79)	1.63 (1.16-2.24)	1.32 (1.26-1.39)	1.25 (1.18-1.33)	1.39 (1.27-1.53)
AOR (95% CI)	1.18 (1.12-1.24)	1.15 (1.08-1.22)	1.23 (1.11-1.35)	1.34 (1.05-1.68)	1.19 (0.86-1.63)	1.53 (1.08-2-13)	1.18 (1.12-1.25)	1.16 (1.09-1.23)	1.22 (1.11-1.35)
**Fragility fracture**									
OR (95% CI)	1.31 (1.24-1.37)	1.22 (1.15-1.30)	1.43 (1.30-1.56)	1.57 (1.27-1.93)	1.41 (1.03-1.89)	1.77 (1.31-2.35)	1.30 (1.23-1.36)	1.21 (1.06-1.20)	1.40 (1.27-1.54)
AOR (95% CI)	1.18 (1.12-1.24)	1.12 (1.05-1.19)	1.28 (1.17-1.41)	1.50 (1.21-1.85)	1.33 (0.97-1.80)	1.70 (1.25-2-27)	1.18 (1.12-1.24)	1.13 (1.06-1.20)	1.26 (1.15-1.39)
With fall^a^									
OR (95% CI)	1.27 (1.19-1.35)	1.23 (1.14-1.32)	1.24 (1.07-1.42)	1.50 (1.02-2.13)	1.38 (0.82-2.19)	1.68 (0.93-2.86)	1.27 (1.19-1.35)	1.22 (1.13-1.31)	1.22 (1.06-1.41)
AOR (95% CI)	1.11 (1.04-1.19)	1.11 (1.03-1.20)	1.05 (0.91-1.21)	1.38 (0.93-1.99)	1.21 (0.71-1.96)	1.68 (0.93-2.88)	1.12 (1.05-1.20)	1.13 (1.05-1.22)	1.05 (0.90-1.22)
**Distal arm fracture**									
OR (95% CI)	1.17 (1.09-1.25)	1.10 (1.01-1.19)	1.26 (1.07-1.48)	1.00 (0.70-1.38)	0.85 (0.51-1.32)	1.22 (0.72-1.96)	1.18 (1.10-1.27)	1.10 (1.02-1.19)	1.27 (1.06-1.50)
AOR (95% CI)	1.09 (1.01-1.17)	1.04 (0.96-1.13)	1.21 (1.02-1.43)	0.94 (0.65-1.31)	0.76 (0.46-1.20)	1.24 (0.73-2.01)	1.10 (1.02-1.19)	1.06 (0.98-1.16)	1.22 (1.02-1.45)
**Vertebral fracture**									
OR (95% CI)	1.73 (1.52-1.96)	1.69 (1.41-2.01)	1,80 (1.50-2.16)	3.26 (2.13-4.88)	3.24 (1.72-5.88)	3.29 (1.84-5.69)	1.63 (1.42-1.86)	1.59 (1.32-1.91)	1.70 (1.20-1.79)
AOR (95% CI)	1.51 (1.33-1.72)	1.47 (1.22-1.76)	1.56 (1.29-1.88)	3.00 (1.95-4.53)	3.11 (1.64-5.68)	3.00 (1.65-5.26)	1.43 (1.25-1.64)	1.40 (1.15-1.69)	1.47 (1.20-1.79)
**Hip fracture**									
OR (95% CI)	1.30 (1.18-1.44)	1.27 (1.12-1.43)	1.31 (1.08-1.58)	4.06 (1.59-9.82)	1.34 (0.20-5.21)	12.37 (3.26-59)	1.29 (1.16-1.43)	1.26 (1.11-1.42)	1.27 (1.05-1.53)
AOR (95% CI)	1.10 (0.99-1.22)	1.08 (0.95-1.23)	1.10 (0.90-1.33)	3.80 (1.46-9.37)	1.17 (0.17-4.83)	11.09 (2.85-53.00)	1.12 (1.01-1.25)	1.12 (0.98-1.27)	1.09 (0.89-1.32)
**Osteoporosis medication**									
OR (95% CI)	1.35 (1.26-1.44)	1.29 (1.20-1.38)	1.30 (1.08-1.55)	1.83 (0.82-3.70)	2.01 (0.73-4.81)	1.53 (0.35-4.85)	1.35 (1.26-1.44)	1.28 (1.19-1.38)	1.31 (1.08-1.56)
AOR (95% CI)	1.09 (1.01-1.16)	1.07 (0.99-1.15)	1.03 (0.85-1.25)	0.94 (0.40-1.98)	1.11 (0.39-2.77)	0.78 (0.17-2.67)	1.11 (1.04-1.20)	1.10 (1.02-1.19)	1.07 (0.88-1.30)

Abbreviations: AOR, adjusted odds ratio; OR, odds ratio.

^a^ On the same level.

There was an association between NFATs and previous fragility fracture (AOR, 1.18; 95% CI, 1.12-1.24), which was also seen in both sexes. Among younger individuals with NFATs, only men exhibited an association with previous fragility fractures (AOR, 1.70; 95% CI, 1.25-2.27). An association with distal arm fracture was found in men with NFATs. Vertebral fractures occurred in 311 patients (1.5%) with NFATs compared with 1115 controls (0.9%). An association between NFATs and vertebral fractures was also found (AOR, 1.51; 95% CI, 1.33-1.72). The association between vertebral fracture and NFATs was particularly strong in younger individuals (AOR, 3.00; 95% CI, 1.95-4.53), whereas the association with hip fracture was found in younger men with NFATs (AOR, 11.09; 95% CI, 2.85-53.00). Details on medications for osteoporosis and fractures with a fall on the same level are given in Table 1 and Table 2.

### New Fractures and Osteoporosis Medications

The association between new fractures and osteoporosis medications was analyzed by means of Cox proportional hazards regression analysis, with and without adjustment for potential confounders (Table 3). During the up to 16 years of follow-up (median [IQR], 4.9 [2.2-8.2] years), new fractures occurred more often in individuals with NFATs than in controls (3127 of 20 390 [15.3%] vs 16 086 of 125 392 [12.8%]; HR, 1.40; 95% CI, 1.34-1.45; adjusted hazard ratio [AHR], 1.27; 95% CI, 1.22-1.33) (Figure, A), with similar associations in both sexes. Among younger patients with NFATs, NFATs were associated with new fractures only in men (AHR, 1.48; 95% CI, 1.20-1.82). In addition, NFATs were associated with new fragility fractures in all groups except younger women. However, the association was strongest among younger men (AHR, 2.13; 95% CI, 1.56-2.91) (Table 3 and Figure, B).

**Table 3.** Fractures and Osteoporosis Medications Analysis in Individuals With Nonfunctioning Adrenal Tumors With Follow-Up Up to 16 Years Compared With Controls

	All				Aged <50 y				Aged ≥50 y		
	All	Women	Men	All		Women	Men	All		Women	Men
Follow-up time, y	4.9 (2.2-8.2)	4.9 (2.2-8.2)	4.8 (2.1-8.2)	5.9 (2.8-9.5)		6.1 (3.1-9.5)	5.8 (2.5-9.5)	4.7 (2.1-8.0)		4.7 (2.1-8.0)	4.7 (2.1-8.0)
**New fractures**											
HR (95% CI)	1.40 (1.34-1.45)	1.33 (1.27-1.39)	1.46 (1.36-1.56)	1.47 (1.28-1.70)		1.34 (1.10-1.63)	1.65 (1.35-2.02)	1.38 (1.32-1.46)		1.33 (1.27-1.39)	1.44 (1.34-1.55)
AHR (95% CI)	1.27 (1.22-1.33)	1.26 (1.20-1.32)	1.31 (1.22-1.40)	1.30 (1.12-1.50)		1.15 (0.94-1.41)	1.48 (1.20-1.82)	1.27 (1.21-1.34)		1.27 (1.21-1.33)	1.32 (1.22-1.42)
With fall^a^											
HR (95% CI)	1.36 (1.29-1.43)	1.32 (1.25-1.40)	1.31 (1.19-1.45)	1.45 (1.14-1.82)		1.34 (1.00-1.81)	1.64 (1.12-2.42)	1.37 (1.30-1.44)		1.33 (1.25-1.40)	1.32 (1.18-1.46)
AHR (95% CI)	1.24 (1.18-1.30)	1.26 (1.19-1.34)	1.19 (1.08-1.32)	1.19 (0.93-1.52)		1.08 (0.79-1.46)	1.48 (0.99-2.20)	1.24 (1.18-1.31)		1.27 (1.19-1.34)	1.19 (1.08-1.32)
**Fragility fracture**											
HR (95% CI)	1.38 (1.32-1.45)	1.31 (1.23-1.38)	1.46 (1.33-1.61)	1.62 (1.29-2.02)		1.14 (0.81-1.60)	2.31 (1.70-3.12)	1.38 (1.32-1.46)		1.31(1.24-1.39)	1.42 (1.28-1.57)
AHR (95% CI)	1.28 (1.21-1.34)	1.26 (1.19-1.34)	1.32 (1.20-1.46)	1.44 (1.14-1.81)		0.97 (0.68-1.38)	2.13 (1.56-2.91)	1.27 (1.21-1.34)		1.27 (1.20-1.35)	1.30 (1.17-1.43)
With fall^a^											
HR (95% CI)	1.32 (1.24-1.40)	1.28 (1.19-1.37)	1.27 (1.11-1.44)	1.36 (0.94-1.98)		0.98 (0.60-1.61)	2.51 (1.38-4.54)	1.33 (1.25-1.42)		1.29 (1.20-1.38)	1.26 (1.10-1.43)
AHR (95% CI)	1.21 (1.14-1.29)	1.23 (1.15-1.33)	1.17 (1.03-1.34)	1.14 (0.77-1.68)		0.78 (0.47-1.30)	2.45 (1.34-4.50)	1.22 (1.14-1.30)		1.25 (1.16-1.34)	1.15 (1.00-1.32)
**Distal arm fracture**											
HR (95% CI)	1.15 (1.05-1.26)	1.09 (0.99-1.20)	1.16 (0.91-1.47)	0.83 (0.55-1.24)		0.80 (0.48-1.32)	0.87 (0.44-1.75)	1.18 (1.08-1.30)		1.10 (0.99-1.22)	1.20 (0.94-1.55)
AHR (95% CI)	1.08 (0.99-1.18)	1.09 (0.99-1.21)	1.01 (0.79-1.28)	0.76 (0.50-1.14)		0.70 (0.42-1.17)	0.87 (0.43-1.75)	1.10 (1.00-1.21)		1.11 (1.00-1.23)	1.06 (0.82-1.37)
**Vertebral fracture**											
HR (95%CI)	1.96 (1.72-2.23)	1.87 (1.57-2.23)	2.10 (1.72-2.55)	2.90 (1.66-5.04)		1.96 (0.79-4.88)	3.83 (1.88-7.79)	1.93 (1.69-2.20)		1.87 (1.57-2.23)	2.04 (1.66-2.50)
AHR (95%CI)	1.83 (1.60-2.09)	1.74 (1.45-2.08)	1.94 (1.58-2.37)	2.61 (1.48-4.61)		2.14 (0.86-5.36)	3.00 (1.44-6.25)	1.80 (1.57-2.07)		1.74 (1.45-2.08)	1.88 (1.52-2.32)
**Hip fracture**											
HR (95% CI)	1.44 (1.33-1.56)	1.43 (1.30-1.57)	1.36 (1.17-1.58)	3.74 (1.65-8.48)		2.16 (0.44-10.7)	4.74 (1.80-12.5)	1.44 (1.33-1.56)		1.43 (1.30-1.57)	1.36 (1.17-1.58)
AHR (95% CI)	1.39 (1.28-1.50)	1.41 (1.28-1.55)	1.32 (1.14-1.54)	2.75 (1.17-6.45)		1.52 (0.31-7.57)	3.76 (1.37-10.30)	1.38 (1.27-1.49)		1.41 (1.28-1.55)	1.30 (1.11-1.51)
**Osteoporosis medications**											
HR (95% CI)	1.66 (1.56-1.76)	1.54 (1.44-1.65)	1.81 (1.59-2.07)	3.10 (2.08-4.62)		2.73 (1.71-4.34)	4.61 (2.07-10.3)	1.65 (1.55-1.76)		1.53 (1.43-1.64)	1.81 (1.58-2.07)
AHR (95% CI)	1.52 (1.43-1.62)	1.47 (1.37-1.58)	1.79 (1.56-2.05)	2.28 (1.50-3.45)		2.06 (1.27-3.34)	3.04 (1.27-7.26)	1.50 (1.41-1.60)		1.45 (1.36-1.56)	1.76 (1.53-2.02)

Abbreviations: AHR, adjusted hazard ratio; HR, hazard ratio.

^a^ On the same level.

**Figure.**
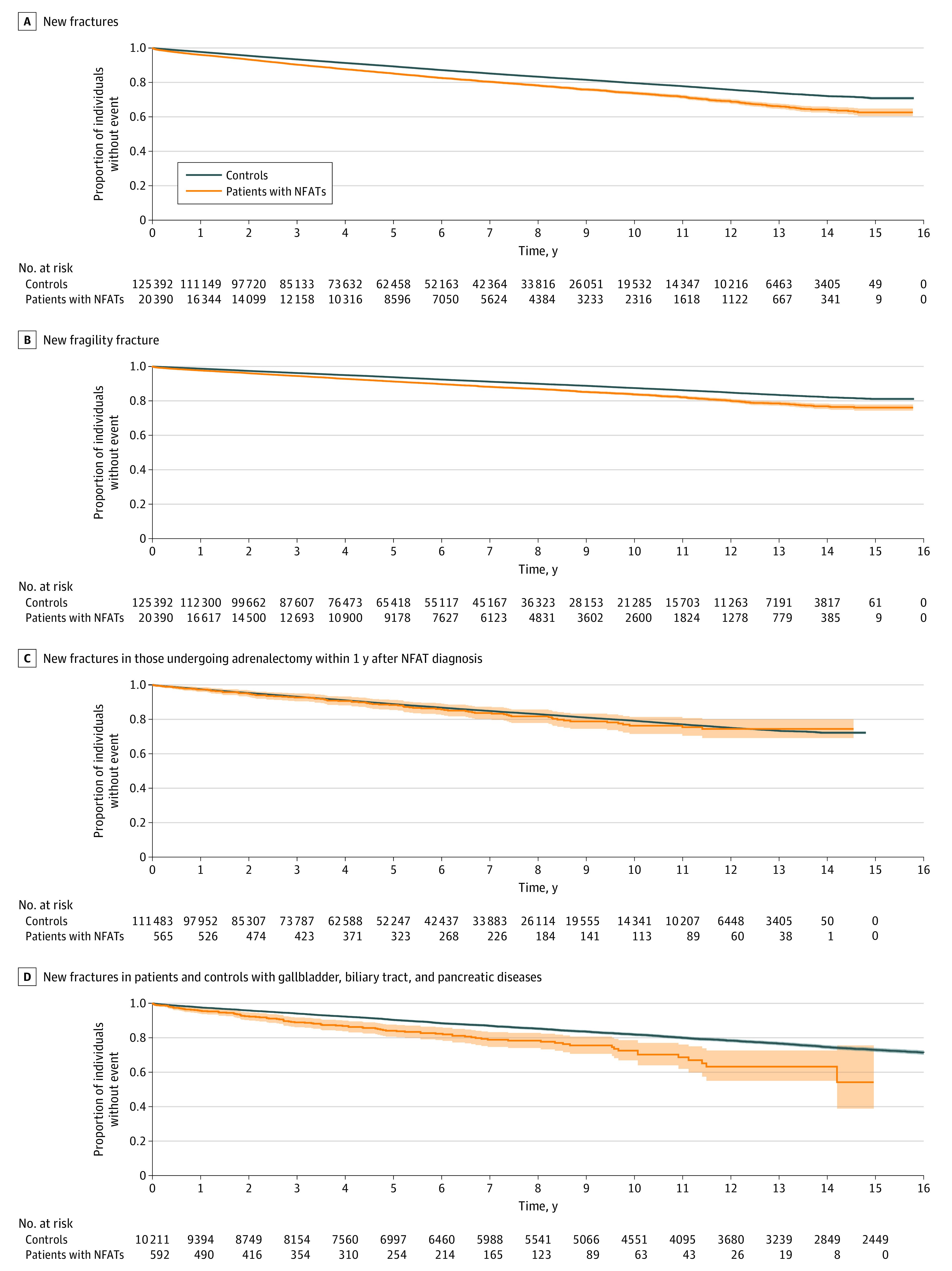
New Fractures and Fracture Fragility Shaded areas indicate 95% CIs; NFAT, nonfunctional adrenal tumor. *P* < .001 for all comparisons.

For both new vertebral and hip fractures, there were associations in all groups, except in younger women with NFATs. The associations were especially strong in younger men with NFATs (vertebral fracture: AHR, 3.00; 95% CI, 1.44-6.25; hip fracture: AHR, 3.76; 95% CI, 1.37-10.30).

New dispensation of medications for osteoporosis was more common in all groups with NFATs compared with controls. There was an association in younger men with NFATs (AHR, 3.04; 95% CI, 1.27-7.26). Details on new fractures with a fall on the same level are given in Table 3.

### Adrenalectomy

Of all 20 390 individuals diagnosed with an NFAT, 593 patients (2.9%), of whom 356 (60.0%) were female, underwent an adrenalectomy within the first year. In this group, the incidences of new fractures (HR, 1.06; 95% CI, 0.86-1.30; AHR, 1.12; 95% CI, 0.90-1.38) (Figure, C) and fragility fractures (HR, 0.93; 95% CI, 0.70-1.24; AHR, 1.10; 95% CI, 0.83-1.46) were no different compared with controls.

### Sensitivity Analyses

Among individuals with gallbladder, biliary tract, and pancreas diseases, the associations between NFATs and previous as well as new fractures remained (OR, 1.68; 95% CI, 1.35-2.06; AOR, 1.48; 95% CI, 1.18-1.84; HR, 1.70; 95% CI, 1.38-2.08; AHR, 1.44; 95% CI, 1.17-1.77) (Figure, D). Similar associations were seen for fragility fracture (OR, 1.94; 95% CI, 1.49-2.50; AOR, 1.67; 95% CI, 1.26-2.19; HR, 1.55; 95% CI, 1.17-2.05; AHR, 1.23; 95% CI, 0.92-1.63). In the 2 remaining sensitivity analyses, the association with fractures was still present and similar to the results in the main analysis (index shifted at 3 months: OR, 1.44; 95% CI, 1.38-1.49; AOR, 1.30; 95% CI, 1.25-1.36; HR, 1.36; 95% CI, 1.30-1.41; AHR, 1.22; 95% CI, 1.17-1.27; index shifted at 12 months: OR, 1.44; 95% CI, 1.39-1.50; AOR, 1.31; 95% CI, 1.26-1.37; HR, 1.33; 95% CI, 1.28-1.39; AHR, 1.18; 95% CI, 1.13-1.23).

## Discussion

This is the largest study, to our knowledge, addressing NFATs and fractures, encompassing 20 390 individuals with NFATs and 125 392 controls. Moreover, it covers all new cases of NFATs in Sweden during 15 years, minimizing the risk of selection bias. We found an increased prevalence of previous fractures when the NFAT was diagnosed but also an increased incidence of new fractures during follow-up. Similar findings were found when restricting the analysis to fragility fractures. Vertebral and hip fractures especially occurred more often in individuals with NFATs than in controls. Men younger than 50 years with an NFAT had a higher relative fracture risk, whereas women in the same age group did not. Adrenalectomy was associated with an apparent normalization of the fracture risk in those with an NFAT, although the statistical power was restricted in this small subgroup.

Previous studies in patients with adrenal tumors have found increased fracture risk in individuals with higher cortisol levels.^11,12,17^ In contrast to the current study, these studies did not exclude individuals with MACS. Moreover, a control group without known adrenal masses is very rare in studies of adrenal tumors. However, Li et al^13^ performed a population-based cohort study of 1004 patients with NFATs and 1004 controls. This study found not only a higher frequency of previous fractures at index but also an increased risk of new fractures during follow-up in individuals with NFATs compared with those without (AHR, 1.27; 95% CI, 1.07-1.52).^18^ These results are very similar to ours (AHR, 1.27; 95% CI, 1.23-1.33). Only 21.1% of our patients had a previous fracture compared with 47.9% in their cohort. However, Li et al^13^ had medical records available since 1966 in their examined county, whereas we had only national data using *ICD-10*, which was introduced in Sweden in 1997 (ie, an almost 20-year difference, which may explain the prevalence difference). Moreover, our vertebral fracture prevalence rate of 1.5% in patients with NFATs (compared with 0.9% in controls) was also much lower than the 6.4% reported by Li et al^13^ but even lower compared with the rates of 22.9% to 24.7% reported by other recent studies.^11,12^ We suspect that this drastic difference in prevalence is due to inclusion of asymptomatic vertebral fractures in the previous studies^11,12^ because all patients with NFATs had their spines evaluated radiologically, whereas our study and the study by Li et al^13^ possibly included only symptomatic vertebral fractures. Moreover, these studies with very high vertebral fracture rates were from tertiary specialized endocrine clinics, likely introducing a selection bias, whereas our study and the study by Li et al^13^ were population based, minimizing the risk of selection bias. Furthermore, we used only *ICD-10* codes, which probably underestimate the prevalence of vertebral fractures, whereas Li et al^13^ reviewed the medical records in detail and had a longer time span when fractures could occur as discussed earlier. However, because we compared patients with NFATs with controls who had been diagnosed according to *ICD-10* codes as well, any underestimation of vertebral fractures was presumably the same in both cases and controls.

Fragility fractures, and especially vertebral fractures, were associated with NFATs. Bone loss and fragility fractures are commonly reported in both exogenous and endogenous glucocorticoid exposure.^17,19^ Fractures due to glucocorticoid excess can occur at any skeletal site; however, they are more prevalent at trabecular sites and especially at the vertebrae.^17,19^ Vertebral fractures, as well as other fragility fractures, can occur in glucocorticoid excess even if BMD is normal.^19^ Moreover, in asymptomatic endogenous cortisol excess, vertebral fracture may be the first sign.^19^ The underlying reason for our findings of more fractures, including fragility fractures in general and vertebral fractures in particular, may be mild glucocorticoid excess. In addition, NFATs may be hormonally inactive, but with time they may commence secreting small amounts of cortisol and thus increase the fracture risk. Cortisol concentrations, however, may already fluctuate from the beginning, making NFATs not so nonfunctional. This hypothesis has already been reported by previous authors.^1^ Furthermore, we did not have the dexamethasone suppression test results available, so some cases of MACS may have been included, inflating the results. If using the percentages (43%-45%) mentioned in previous studies,^6,7,8^ many of our patients with NFATs may have had MACS. However, it has been reported that an *ICD-10* code of Cushing syndrome overestimates overt Cushing syndrome in register-based research^20^; thus, many individuals diagnosed with Cushing syndrome by an *ICD-10* code may in reality have MACS.^21^ Moreover, previous studies of NFATs (confirmed by dexamethasone suppression tests) found increased occurrence of type 2 diabetes^1^ and increased intima media thickness,^22^ indicating slight overproduction of cortisol not detected by dexamethasone suppression tests. Additionally, patients with NFATs may undergo more frequent follow-up, including radiologic examinations, which could identify fractures and introduce detection bias. This, however, still cannot explain the increased hip fracture rate because these fractures most likely will result in admission and surgery. Another possible explanation may be that the NFAT was found due to trauma and the fractures were part of the trauma or that the individual was prone to trauma (eg, by alcohol misuse). However, only a few adrenal tumors are found while investigating traumatic injuries (approximately 1.5%),^23^ and fractures associated with a fall on the same level (ie, low-energy trauma) were also increased in the current study. We also adjusted for alcohol misuse, although mild-moderate alcohol misuse may be underestimated by using *ICD-10* codes. In addition, vertebral fracture can result from activities such as coughing and carrying things or occur spontaneously in individuals with osteoporosis.^24^ Increased fracture prevalence has also been found in patients with primary aldosteronism, especially vertebral fractures.^25^ Even though overt primary aldosteronism can be assumed to have been excluded in the current study, mild aldosteronism, especially if not resulting in hypertension or hypokalemia, may have been included and may have affected the fracture risk. Finally, other hitherto undetected factors that coincide with NFATs may contribute to the increased fracture risk.

Adrenalectomy reduces the vertebral fracture risk in patients with mild endogenous glucocorticoid excess.^26^ Because the patients with NFATs who had undergone adrenalectomy in the current study also appeared to have normalized fracture incidence, it could be speculated that mildly unphysiologic cortisol concentrations became physiologic, leading to fewer fractures. However, randomized clinical studies are required to confirm this hypothesis. Moreover, even though the number of patients with adrenalectomy was reasonable (n = 593), the number of fractures during follow-up may have been too few to demonstrate a significant increased fracture rate. Even though adrenalectomy in patients with NFATs is mainly performed when there is a suspected adrenal malignant tumor and the general health should not play a major part in the decision, the patients accepted for adrenalectomy may still be healthier, thus introducing a selection bias.

Interestingly, we found a relatively more pronounced risk in men younger than 50 years, whereas younger women did not seem to have any association between NFATs and fragility fractures at all. There were both increased prevalence of vertebral and hip fractures and increased incidence of the same fracture type in younger men. An increased risk of vertebral fractures has been seen in men compared with women with NFATs.^11^ The explanation is somewhat unclear. Women’s risk of fractures increases after menopause, and premenopausal women with NFATs may be protected by their eugonadism, but this cannot be the case for men. Fractures in men are strongly associated with estradiol concentrations^27^; however, whether NFATs and estradiol are associated is unclear.

We could not match our controls for imaging because we did not have data on who had undergone imaging. However, by conducting a sensitivity analysis focusing on a group of disorders in which prediagnostic imaging could be presumed in both cases and controls (ie, cases and controls with gallbladder, biliary tract, and pancreas diseases), we could support our results further. In the analysis, both previous fractures and fragility fractures as well as new fractures and fragility fractures were increased compared with controls, and the increase was even more pronounced than in the main analysis. Moreover, we speculated that some patients who underwent imaging did so in the workup of suspected cancer or severe illness, and the NFAT found on computed tomography could be a metastasis of a not yet diagnosed cancer. To avoid the inclusion of seriously ill patients with increased fracture risk, we shifted the index date of the NFAT diagnosis 90 and 365 days in sensitivity analyses, effectively excluding anyone who died or was diagnosed with cancer shortly after the NFAT diagnosis. This approach did not change the results. Overall, in all sensitivity analyses, fracture prevalence and incidence were still significantly increased in individuals with NFATs.

The European Society of Endocrinology clinical guidelines on the management of adrenal incidentalomas suggest against repeated hormonal workup in individuals with NFATs and MACS and recommend yearly clinical follow-up by a general practitioner in patients with MACS not undergoing adrenalectomy.^3^ In contrast, the American Association of Endocrine Surgeons guidelines recommend hormonal reevaluation at a 2- to 5-year interval.^28^ The findings from the current study may suggest follow-up in patients with NFATs as well, especially in younger men with additional risk factors for fractures in which BMD with vertebral fracture assessment may be indicated. However, future prospective trials assessing BMD with vertebral fracture assessment in patients with NFATs, especially younger men, are needed.

### Limitations

The current study has some limitations. Unfortunately, neither hormonal evaluation of the patients nor radiologic reports were available. Accordingly, some people could have been diagnosed with NFATs despite mildly pathological dexamethasone suppression test results. Moreover, because we did not have radiologic reports available, we could not evaluate the Hounsfield units or the adrenal tumor size. Higher Hounsfield units and larger adrenal tumor size may indicate a malignant neoplasm and adrenocortical cancer usually produces cortisol,^3,29^ whereas larger size in benign adrenal tumors was not associated with increased fracture risk.^11^ Furthermore, we did not have data on race available, which may affect the fracture risk; however, most individuals in Sweden are White, especially in the older population, who were overrepresented in the current study. We could not match imaging in cases and controls, but in one of the sensitivity analyses, we analyzed a group of individuals in whom prior imaging could be assumed in both cases and controls, and the fracture risk was still elevated in patients with NFATs. The imaging that resulted in the NFAT diagnosis may have been done due to suspicion of cancer, and in order to remove all cases of malignant neoplasm or death occurring just after the NFAT diagnosis, 2 more sensitivity analyses were performed shifting the index date 3 and 12 months, but the fracture results remained. Finally, although we adjusted for a broad range of potential confounders that could result in elevated fracture risk, residual confounding cannot be excluded.

## Conclusions

This study found that NFATs are associated with an increased prevalence and incidence of fractures, including fragility fractures. The association between NFATs and fractures is most pronounced among younger men. Those who underwent adrenalectomy had normalized fracture incidence. Therefore, patients with NFATs, particularly younger men, should have bone health evaluation with appropriate treatment and monitoring. Long-term follow-up seems indicated.

## Notes

Supplement 1. **eTable 1.** Fractures, Falls, Potential Confounders and Other Conditions Based on ICD Diagnoses From the National Patient Registry Including Both In- and Outpatient Care and ATC Codes From the Swedish Prescribed Drug Register **eTable 2.** Comorbidities Affecting Bone Mineral Density and Different Fractures by Sex Among Cases With Non-Functioning Adrenal Tumor and Controls at Baseline **eFigure.** The Age Distribution at Baseline of Included Patients With Non-Functional Adrenal Tumors (Dark) and Controls (Grey)

Supplement 2.
Data Sharing Statement
