# Supplementary material for: Prevalence and Incidence of Fractures in Patients With Nonfunctional Adrenal Tumors
Source: JAMA Netw Open. 2024 Apr 15;7(4):e246453. doi: 10.1001/jamanetworkopen.2024.6453 (PMC11019395; doi:10.1001/jamanetworkopen.2024.6453)
Supplement: Supplement 1. — eTable 1. Fractures, Falls, Potential Confounders and Other Conditions Based on ICD Diagnoses From the National Patient Registry Including Both In- and Outpatient Care and ATC Codes From the Swedish Prescribed Drug Register eTable 2. Comorbidities Affecting Bone Mineral Density and Different Fractures by Sex Among Cases With Non-Functioning Adrenal Tumor and Controls at Baseline eFigure. The Age Distribution at Baseline of Included Patients With Non-Functional Adrenal Tumors (Dark) and Controls (Grey) [file jamanetwopen-e246453-s001.pdf]

Supplementary Online Content

Lindh JD, Patrova J, Mannheimer B, Falhammar H. Prevalence and incidence of fractures in patients with nonfunctional adrenal tumors. *JAMA Netw Open*. 2024;7(4):e246453. doi:10.1001/jamanetworkopen.2024.6453

eTable 1. Fractures, Falls, Potential Confounders and Other Conditions Based on ICD Diagnoses From the National Patient Registry Including Both In- and Outpatient Care and ATC Codes From the Swedish Prescribed Drug Register

eTable 2. Comorbidities Affecting Bone Mineral Density and Different Fractures by Sex Among Cases With Non-Functioning Adrenal Tumor and Controls at Baseline

eFigure. The Age Distribution at Baseline of Included Patients With Non-Functional Adrenal Tumors (Dark) and Controls (Grey).

This supplementary material has been provided by the authors to give readers additional information about their work.

© 2024 Lindh JD et al. *JAMA Network Open.*

eTable 1. Fractures, falls, potential confounders and other conditions based on ICD diagnoses from the National Patient Registry including both in- and outpatient care as well as and ATC codes from the Swedish Prescribed Drug Register

	ICD-10 or ATC code
**Primary outcome**	
Any fracture	S02, S12, S22, S32, S42, S52, S62, S72, S82, S92, T02, T08, T10, T12, T14.2
**Secondary outcomes**	
Any fracture with fall on the same level	S02, S12, S22, S32, S42, S52, S62, S72, S82, S92, T02, T08, T10, T12, T14.2 AND W00, W01, W03, W04, W05, W06, W07, W08, W18
Fragility fracture	S12, S22.0, S22.1, S32.0, S32.7, T02.1, T08, S42, S52.5, S52.6, S72, T10
Fragility fracture with fall on the same level	S12, S22.0, S22.1, S32.0, S32.7, T02.1, T08, S42, S52.5, S52.6, S72, T10 AND W00, W01, W03, W04, W05, W06, W07, W08, W18
Distal arm fracture	S52.5, S52.6
Vertebral fracture	S12, S22.0, S22.1, S32.0, S32.7, T02.1, T08
Hip fracture	S72.0, S72.1, S72.2
Osteoporosis medications	M05BA, M05BB, M05BX04, M05BX06, H05AA
**Confounders**	
COPD	J44, J43
Inflammatory bowel disease	K51, K50
Celiac disease/malabsorption	K90
Alcohol misuse	E24.4, F10, G62.1, G72.1, I42.6, K29.2, K70, K86
Primary hyperparathyroidism	E210
Hyperthyroidism	E05
Vitamin D deficiency	E55
Malnutrition	E43.9, E41.9
Hypogonadism[a]	E29.1, E23.0E, E23.0F, E28.3
Diabetes mellitus	E10-E14
Glucocorticoid usage >90 days	H02AB
Previous fracture[b]	S02, S12, S22, S32, S42, S52, S62, S72, S82, S92, T02, T08, T10, T12, T14.2
**Other interventions/diagnosis**	
Adrenalectomy	BCA20-BCA41
Gallbladder/biliary tract/pancreas diseases	K80-87

[a]Not including normal menopause. [b]Only used when analyzing adjusted hazard ratio. COPD, chronic obstructive pulmonary disease.

© 2024 Lindh JD et al. *JAMA Network Open.*

eTable 2. Comorbidities affecting bone mineral density and different fractures by sex among cases with non-functioning adrenal tumor and controls at baseline.

	Female cases (n=12,120)	Female controls (n=69,994)	Male cases (n=8,270)	Male controls (n=55,398)
Age (yrs)	66 (57;73)	66 (57;74)	65 (57;72)	66 (58;73)
**Diagnosis**				
COPD	685 (5.7%)	1923 (2.7%)	628 (7.6%)	1466 (2.6%)
Inflammatory bowel disease	274 (2.3%)	754 (1.1%)	145 (1.8%)	630 (1.1%)
Celiac disease/malabsorption	64 (0.5%)	332 (0.5%)	33 (0.4%)	154 (0.3%)
Alcohol misuse	406 (3.3%)	1111 (1.6%)	600 (7.3%)	2134 (3.9%)
Hyperparathyroidism	191 (1.6%)	373 (0.5%)	51 (0.6%)	78 (0.1%)
Hyperthyroidism	315 (2.6%)	1131 (1.6%)	63 (0.8%)	227 (0.4%)
Vitamin D deficiency	12 (0.1%)	63 (0.0.9%)	4 (0.05%)	31 (0.06%)
Malnutrition	8 (0.07%)	16 (0.02%)	2 (0.02%)	10 (0.02%)
Hypogonadism[a]	12 (0.1%)	42 (0.06%)	29 (0.4%)	107 (0.2%)
Diabetes mellitus	1601 (13.2%)	4345 (6.2%)	1416 (17.1%)	5315 (9.6%)
**Previous fractures**	2778 (22.9%)	12,944 (18.5%)	1532 (18.4%)	7379 (13.3%)
with fall on the same level	1615 (13.3%)	76634 (10.9%)	593 (7.2%)	2890 (5.2%)
Fragility fracture	1542 (12.7%)	7453 (10.6%)	623 (7.5%)	2995 (5.4%)
with fall on the same level	988 (8.2%)	4718 (6.7%)	245 (3.0%)	1335 (2.4%)
Distal arm fracture	776 (6.4%)	4107 (5.9%)	176 (2.1%)	939 (1.7%)
Vertebral fracture	160 (1.3%)	551 (0.8%)	151 (1.8%)	564 (1.0%)
Hip fracture	323 (2.7%)	1480 (2.1%)	133 (1.6%)	682 (1.2%)
**Medications**<br>Glucocorticoids > 90 d	1710 (14.1%)	6366 (9.1%)	775 (9.4%)	3652 (6.5%)
Osteoporosis medications	1022 (8.4%)	4663 (6.7%)	143 (1.7%)	741 (1.3%)

[a]Not including normal menopause. COPD, chronic obstructive pulmonary disease.

© 2024 Lindh JD et al. *JAMA Network Open.*

eFigure 1. The age distribution at baseline of included patients with non-functional adrenal tumors (dark) and controls (grey).

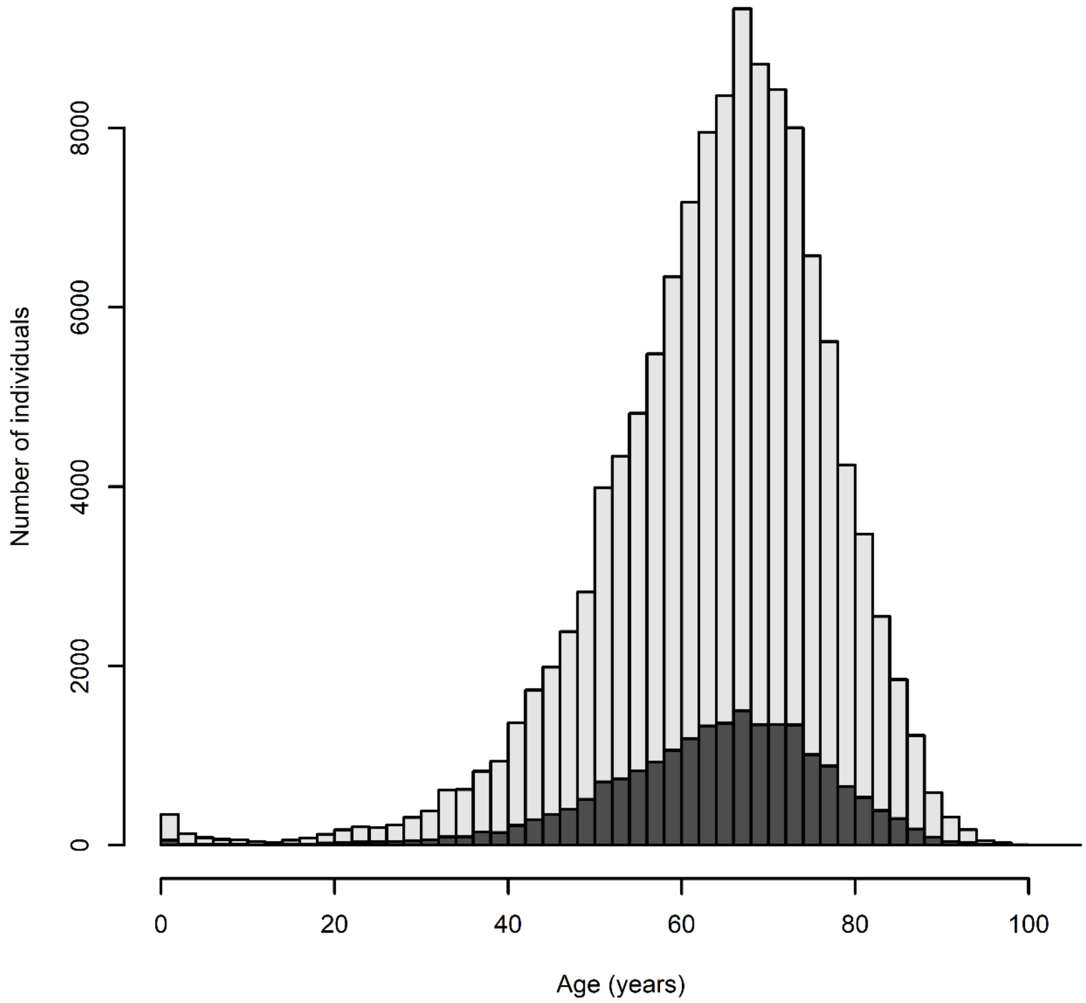

© 2024 Lindh JD et al. *JAMA Network Open.*
