# Supplementary material for: Prevalence and Incidence of Fractures in Patients With Nonfunctional Adrenal Tumors
Source: JAMA Netw Open. 2024 Apr 15;7(4):e246453. doi: 10.1001/jamanetworkopen.2024.6453 (PMC11019395; doi:10.1001/jamanetworkopen.2024.6453)
Supplement: Supplement 2. — Data Sharing Statement [file jamanetwopen-e246453-s002.pdf]

Data Sharing Statement

Lindh. Prevalence and Incidence of Fractures in Patients With Nonfunctional Adrenal Tumors. *JAMA Netw Open*. Published April 15, 2024. doi:10.1001/jamanetworkopen.2024.6453

Data
Data available: No

Additional Information
Explanation for why data not available: We will consider sharing deidentified, individual participant-level data that underlie the results reported in this Article on receipt of a request detailing the study hypothesis and statistical analysis plan. All requests should be sent to the corresponding author. The corresponding author and lead investigators of this study will discuss all requests and make decisions about whether data sharing is appropriate based on the scientific rigor of the proposal. All applicants will be asked to sign a data access agreement.
